# Development of number line estsimation in Chinese preschoolers: a comparison between numerical and non-numerical symbols

**DOI:** 10.3389/fpsyg.2024.1412151

**Published:** 2024-06-21

**Authors:** Mengxia Li, Jiahui Yang, Xiaohan Lei

**Affiliations:** Department of Psychology, School of Teachers Education, Huzhou University, Huzhou, China

**Keywords:** magnitude representation, number line estimation, non-numerical symbols, numerical symbols, Chinese preschoolers

## Abstract

To examine the level of number line estimation (NLE) in Chinese children with respect to representations of both numerical (Arabic numerals) and non-numerical symbols (dots), a total of 192 Chinese preschoolers aged between 4 and 5 years participated in four different NLE tasks. These tasks were paired to evaluate the accuracy and patterns of children’s estimations in both numerical and non-numerical symbol contexts. Our findings indicate that, for Chinese preschoolers, relatively precise numerical symbol representations begin to emerge as early as 4 years of age. The accuracy of number line estimates for both 4- and 5-year-old children gradually increases in tasks involving both numerical and non-numerical symbols. Additionally, the development and patterns observed in the number line estimates of 4- and 5-year-old Chinese preschoolers are similar in both numerical symbol and non-numerical symbol tasks. These results indicate that the initiation of relatively precise numerical symbol representation and the turning point in the developmental trajectory, where the relatively precise representation for numerical symbols surpasses that of non-numerical ones, occur earlier in Chinese children than in their Western counterparts.

## Introduction

Magnitude representation skills, a foundational cognitive function, play a critical role in individual achievements across various domains, including academic success (Siegler and Braithwaite, 2017), occupational attainment (Geary, 2000; Ritchie and Bates, 2013), and financial wellbeing (Agarwal and Mazumder, 2013). More specifically, a large number of studies have argued that magnitude representations are the core of numerical development (Booth and Siegler, 2006, 2008; Link et al., 2014; Siegler, 2016; Georges et al., 2017; Tam et al., 2019). Magnitude representations have been extensively studied as a predictor of mathematical performance (Libertus et al., 2011; Bonny and Lourenco, 2013; Schneider et al., 2018; Seitz and Weinert, 2022).

Magnitude representation can manifest in both non-numerical symbol (e.g., dots) and numerical symbol (e.g., Arabic numerals) forms (Siegler and Booth, 2005; Ebersbach and Erz, 2014). Some research studies suggest that the magnitude representation of non-numerical and numerical symbols share the same underlying cognitive mechanisms. Both non-numerical and numerical symbols can be processed spatially (for review and discussion, see Patro and Haman, 2012; Toomarian and Hubbard, 2018). Magnitude values can be mapped to a spatial range, such as a line, especially in the form of a horizontal mental number line that extends from left to right (Dehaene and Cohen, 2007).

The performance of number line estimation (NLE) is widely used as an indicator of the basic mental number line and its development in children (Siegler and Opfer, 2003; Barth and Paladino, 2011; Barth et al., 2016). Two typical tasks in NLE are the number-to-position (NP) task and the position-to-number (PN) task. Most of the published research studies on NLE have employed the NP task, with only a few studies incorporating the PN task (cf. Madden, 1966; Davis, 1973; Siegler and Opfer, 2003; Iuculano and Butterworth, 2011; Ashcraft and Moore, 2012). It is noteworthy that young children exhibited distinct representation patterns in the PN and NP tasks within the same number line range (Siegler and Opfer, 2003).

Remarkably, among the hundreds of conducted NLE studies, only a handful of studies utilized non-numerical symbols (e.g., dots). However, some researchers question whether NLE, particularly the representation of numerical symbols, can effectively demonstrate the scaling of magnitude representation (Sasanguie and Reynvoet, 2013; Haman and Patro, 2022). This skepticism arises because the symbolic NLE itself is an outcome of children’s mathematical education. A more revealing aspect might be that, prior to formal mathematics education, children utilize non-numerical symbol representations of magnitude, referred to as the approximate number system (ANS; Haman and Patro, 2022). Given that the ANS is an intuitive and innate system, scholars have suggested that it forms the basis for learning symbolic numerals (Fazio et al., 2014). Therefore, to gain a comprehensive understanding of children’s magnitude representation, it is necessary to investigate their non-numerical symbol representation ability.

Children under the age of 4 years indeed have limitations in precision and numerical range regarding both non-numerical and numerical symbol representations. The ANS allows children to make rough estimates of larger magnitude, while subitizing enables them to quickly and accurately recognize magnitudes of 4 or fewer. For relatively precise magnitude representation greater than 4, some research studies have proposed that non-numerical symbol representation emerges as early as 4 years, while numerical symbol representation emerges approximately at the age of 5 years (Gilmore et al., 2007; Libertus et al., 2011; Kolkman et al., 2013; Toll et al., 2015; Li et al., 2017, 2018). For example, Li et al. (2018) employed a non-numerical symbol comparison task, where children were required to quickly determine which of the two sets of dots contained more without counting them. The results showed that 4-year-old children were capable of estimating non-numerical comparisons but not numerical symbol comparisons; similarly, 5-year-olds exhibited better performance in non-numerical symbols than in numerical symbols. Given that the development of early number cognition relies on cultural, educational, and task factors and the development of NLE among Chinese children surpassed that of Western children (Siegler and Mu, 2008; Göbel et al., 2011; Muldoon et al., 2011; Xu et al., 2013; Laski and Yu, 2014; Dowker and Li, 2019; TIMSS, 2019), the question arises as to whether relatively precise representation of numerical symbol for a magnitude greater than 4 does not emerge until the age of 5 years in Chinese children.

Furthermore, accuracy in the NLE task increases with age (Booth and Siegler, 2006, 2008; Zang et al., 2019; Jung et al., 2020). Studies have found that, depending on the version of the task, adult participants either overestimated or underestimated the spatial magnitude corresponding to numbers (Karolis et al., 2011; Cohen and Ray, 2020). It is unclear whether children’s patterns are overestimated or underestimated in different NLE tasks. Therefore, the second question that remains is whether the patterns of Chinese children’s NLE are overestimated or underestimated and whether the pattern for numerical symbols is similar to that for non-numerical symbols.

The present study employed the NP task and the corresponding PN task, the dots-to-position (DP) task, and the corresponding position-to-dots (PD) task for dot/dots to test both numerical and non-numerical symbol representations in 4- and 5-year-olds. We hypothesized that a relatively precise representation of a numerical symbol for a magnitude greater than 4 will emerge as early as 4 years in Chinese preschoolers, the accuracy of the 4- and 5-year-old children’s NLE will gradually increase in both numerical and non-numerical symbol NLE tasks, and the pattern of the 4- and 5-year-old children’s NLE will be similar in numerical and non-numerical symbol tasks.

## Methods

### Participants

An initial sample of 200 4- to 5-year-old Chinese preschoolers (4-year-old children: 100 and 5-year-old children: 100) were recruited from two kindergartens in China. This study excluded the preschoolers who did not complete all the tasks, and the final sample consisted of 192 children (4-year-old children: 98 and 5-year-old children: 94; of which, 102 were girls and 90 were boys). All preschoolers had learned to recognize numbers and dots up to 10 from their family or kindergarten. Parental consent was obtained prior to testing.

### Materials

Preschoolers were presented with an A4-sized booklet, with a home page used to record the children’s basic information, including name, age, and class. Each of the remaining pages presented a single testing task per page. On each horizontally oriented page, there is a horizontal 23-cm-long line centered on the page. The left end of each line was marked with “0,” and the right end of each line was marked with “10.” Additionally, 2 cm above the center of each number line, a circle was printed. The circle contained either a number from 1 to 9 (inclusive) or 1 to 9 dots (inclusive) or was left blank for preschoolers to fill in the number, or the amount of dots they estimated (varied according to the tasks). Each child completed the NP, PN, DP, and PD tasks, and the order of the four tasks was balanced among the preschoolers. In each task, the estimated number of dots ranged from 1 to 9, with a total of nine numbers or dots, each repeated twice. All the target numbers and dots were sorted in random order.

**Figure 1:**
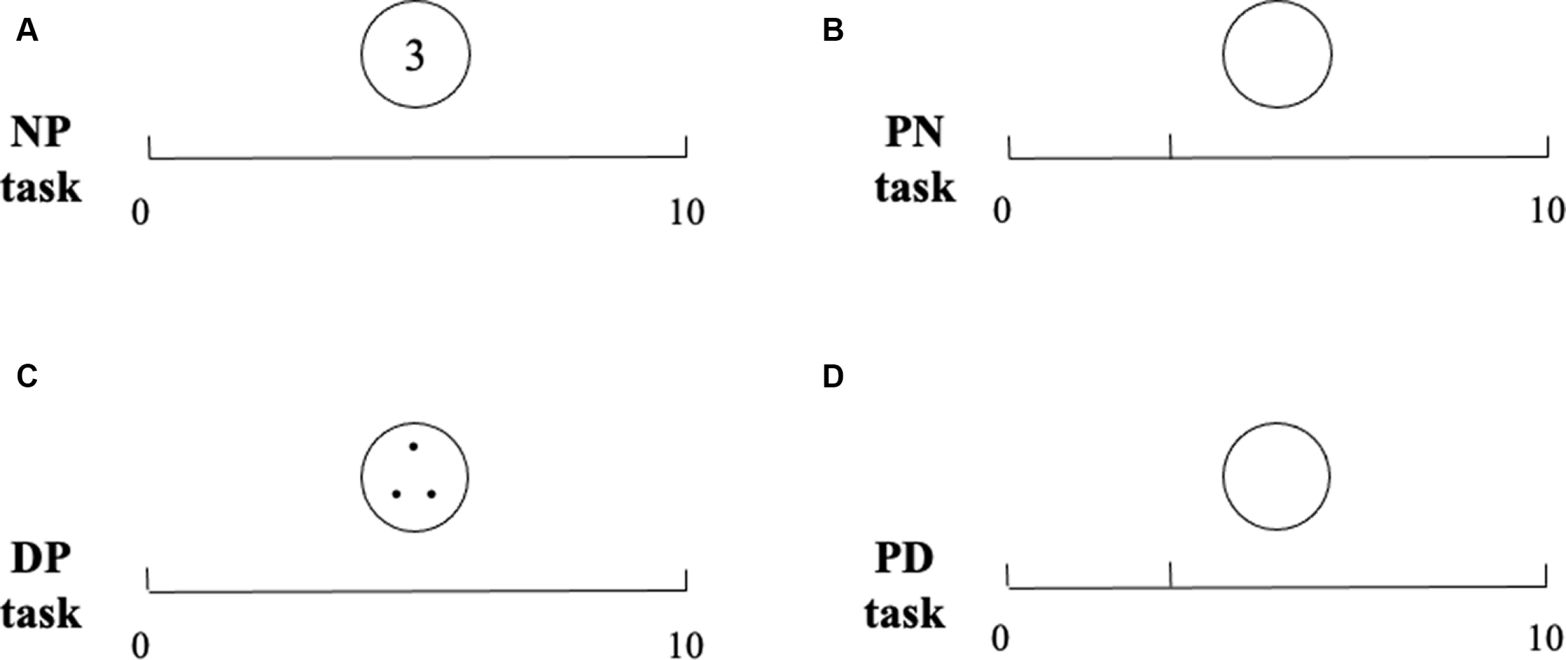
Four NLE tasks.

For the NP task, the preschoolers were asked to mark the position of the target number with a vertical hash mark on the number line. For the PN task, the target number position on the number line was marked by a 1.3-cm vertical hash mark, the preschoolers were asked to report the number of the target number position, and the experimenter filled it in the blank circle above the center of the number line (similar stimuli and designs, see Booth and Siegler, 2006; Slusser and Barth, 2017). Correspondingly, for the DP task, the target dots were printed in a circle, and the preschoolers were asked to mark the position of the target number with a vertical hash mark on the number line. For the PD task, the target number position on the number line was marked by a 1.3-cm vertical hash mark, and the preschoolers were asked to fill in the dots of the target number position in the blank circle above the center of the number line (see Figure 1).

### Data analysis

#### The accuracy of the number line estimation

To analyze the accuracy of the NLE, we calculated the percent absolute error (PAE; PAE =∣Estimate number–Target number∣ /Scale × 100) recommended in previous studies (Siegler and Booth, 2004; Zang et al., 2019).

#### The patterns of the number line estimation

To indicate the patterns of overestimation or underestimation, we also calculated the percent relative estimation error [PRE; PRE = (Estimation number – Target number)/Scale × 100], following the previous studies (Jung et al., 2020). A positive value indicates overestimation, whereas a negative value indicates underestimation.

## Results

### Accuracy of estimation

**Figure 2:**
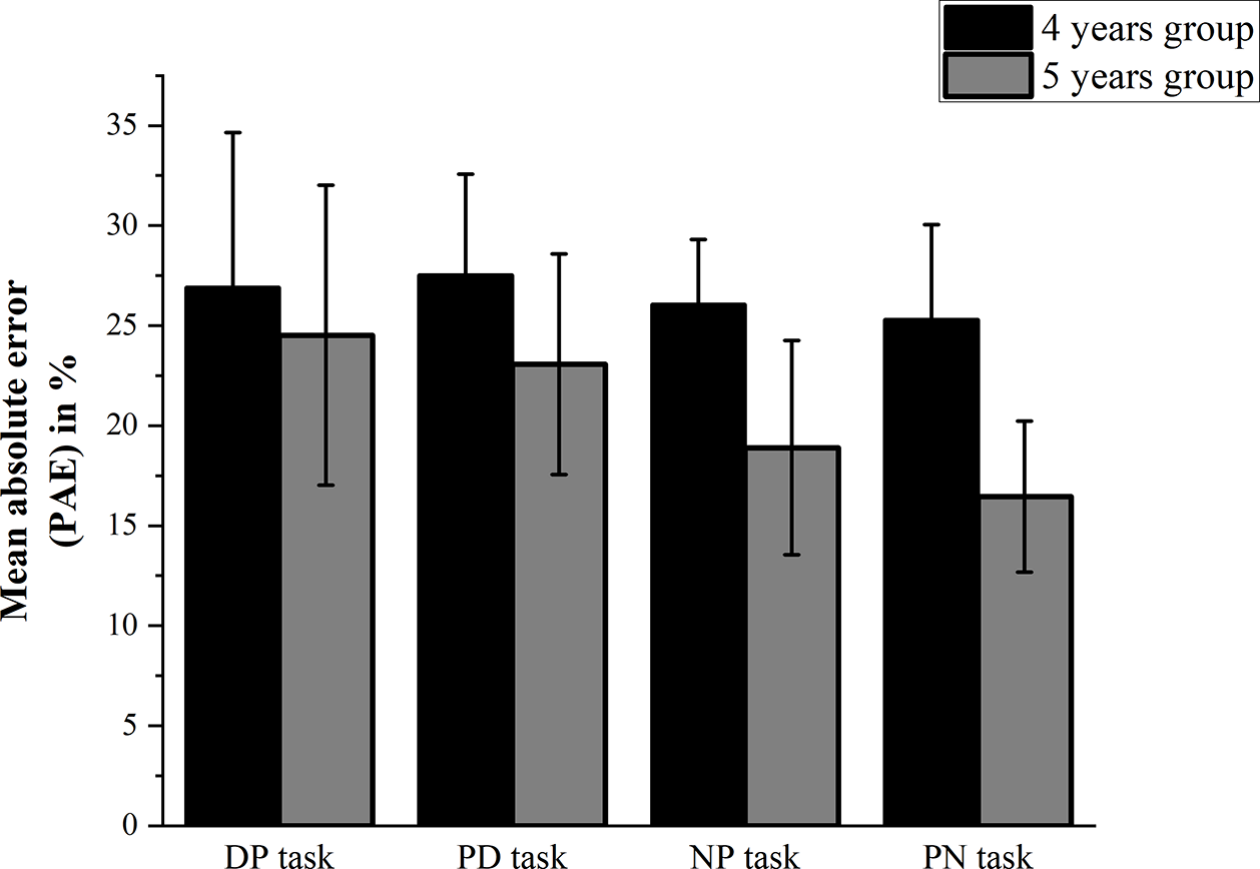
PAE of the four NLE tasks.

Table 1 presents the mean PAE in the four tasks of all preschoolers separately for the two age groups. A 2 (age group: 4 years old vs. 5 years old) × 4 (task: DP task vs. PD task vs. NP task vs. PN task) mixed-design analysis of variance (ANOVA) on the mean PAE revealed a main effect for the task [*F*(1, 3) = 12.49, *p* < 0.001, $\eta_{p}^{2}$ = 0.73], a main effect for age group [*F*(1, 90) = 37.49, *p* < 0.001, $\eta_{p}^{2}$ = 0.70], and a significant interaction between the task group and age group [*F*(3, 270) = 3.84, *p* = 0.034, $\eta_{p}^{2}$ = 0.45]. *Post-hoc* test showed that the mean PAE in the DP task (25.71 ± 1.79) was larger than that in the NP task (22.48 ± 1.04) (*t* = 2.70, *p* = 0.009), and the mean PAE in the PD task (25.29 ± 1.20) was larger than that in the PN task (20.88 ± 1.01) (*t* = 5.28, *p* < 0.001); the mean PAEs in the DP task and the PN task, as well as in the PD task and the NP task, showed no significant differences (*p* > 0.050); the mean PAE of the group of 5-year-olds (20.74 ± 0.67) was less than that of the group of 4-year-olds (26.45 ± 0.67; see Figure 2). Furthermore, simple effect analysis revealed that the mean PAEs of the group of 4-year-olds were significantly larger in the NP and PN tasks than those of the group of 5-year-olds (each *p* < 0.05), and the mean PAE of the group of 4-year-olds was marginally significantly larger in the PD task than that of the group of 5-year-olds (*p* = 0.082).

**Table 1:** Mean percentage absolute error (PAE) in four tasks from two age groups.

Age	Tasks	n	*M* (%)	*SD*
4	DP task	98	26.91	7.75
	PD task	98	27.52	5.06
	NP task	98	26.06	3.25
	PN task	98	25.30	4.75
5	DP task	94	24.52	7.50
	PD task	94	23.07	5.51
	NP task	94	18.90	5.35
	PN task	94	16.45	3.77

Furthermore, simple effect analysis also revealed that the mean PAEs of both 4- and 5-year-olds were larger in the PD task than in the PN task (each *p* < 0.05), and for 5-year-olds, the mean PAE was larger in the DP task than in the NP task (*p* < 0.05). These results suggest that children as young as 4 years old can perform numerical symbol representations, and both 4- and 5-year-olds performed better at numerical compared to non-numerical symbols.

### Patterns of estimation

**Figure 3:**
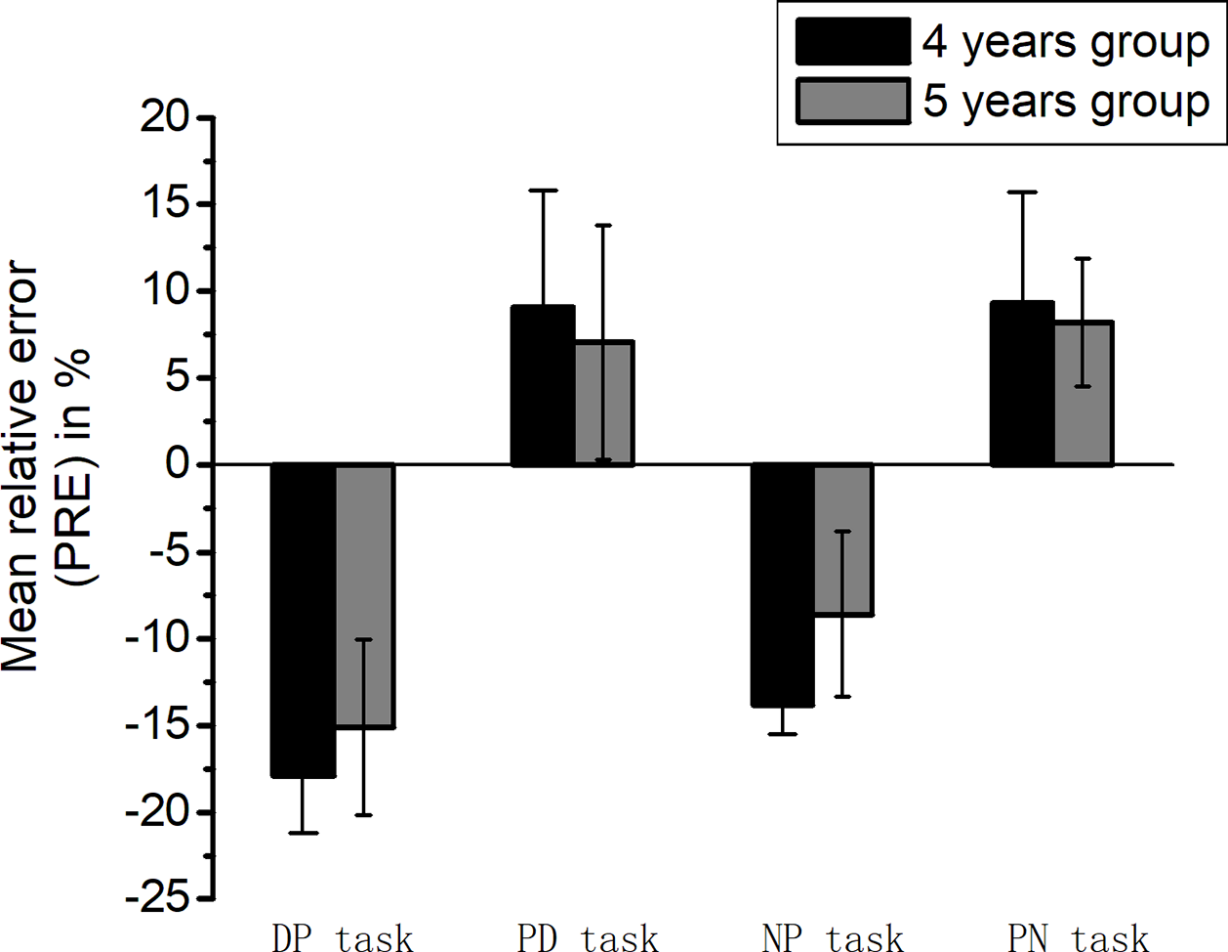
PRE of the four NLE tasks.

A 2 (age group: 4 years old vs. 5 years old) × 4 (task: DP task vs. PD task vs. NP task vs. PN task) analysis of variance on the mean PRE revealed only a significant difference of main effect for the task [*F*(1, 3) = 51.18, *p* < 0.001, $\eta_{p}^{2}$ = 0.92]. Importantly, the mean PRE of the DP task (−16.49 ± 3.02) was lower than that of the NP task (−11.21 ± 2.51) (*t* = −5.28, *p* = 0.003). There was no significant difference in mean PRE between the PN (8.75 ± 3.67) and PD tasks (8.06 ± 4.76) (*t* = 0.69, *p* = 0.704). These results indicate that both the DP and NP tasks exhibited a trend of underestimation, with the DP tasks showing a greater degree of underestimation, while both the PN and PD tasks exhibited a trend of overestimation (see Figure 3). These results showed that both 4- and 5-year-olds performed better at numerical symbols compared to non-numerical symbols. More importantly, children in both age groups have a similar estimation pattern for both numerical and non-numerical symbols.

## Discussion

With the aim of providing more comprehensive developmental trajectories of numerical and non-numerical symbol representations in Chinese preschoolers, we investigated two issues in our study: whether numerical symbol representation emerges by the age of 5 years in Chinese preschoolers and whether the pattern of NLE for numerical symbol representation is similar to that for non-numerical symbol representation in Chinese preschoolers.

Consistent with the hypotheses, we observed that relatively precise representation of numerical symbols for a magnitude greater than 4 emerges as early as 4 years of age in Chinese preschoolers. The accuracy of the NLE for 4- and 5-year-old Chinese preschoolers gradually increases in both numerical symbol and non-numerical symbol tasks, and the pattern of NLE is similar for both types of symbols among these children.

The present study confirmed that children as young as 4 years could relatively precisely represent both non-numerical and numerical symbols of magnitude ranging from 1 to 10. For non-numerical symbol representation, previous studies have assessed the same ability in 4-year-old children. Libertus et al. (2011) measured the ANS of American children using a non-numerical symbol comparison task with a number range from 4 to 15. The results demonstrated that children as young as 4 years were able to represent non-numerical symbols relatively precisely. Similar results were found in 4-year-old children for a broader range of numbers. For example, Li et al. (2018) assessed Chinese children’s representation of non-numerical symbols for numbers ranging from 5 to 50 using the same comparison task, while Wagner and Johnson (2011) tested the children’s relatively precise representation of non-numerical symbols within the range of 1–50. A similar study was carried out by Toll et al. (2015), who even verified that 4-year-old children have the ability to represent numbers within the range of 1–100. The current study was consistent with previous studies in providing evidence for the early development of non-symbolic abilities in children as young as 4 years old.

The current study also indicates that 4-year-old children have the same ability to relatively precisely represent magnitudes greater than 4 using both non-numerical and numerical symbols. Previous studies found that the abilities of Western children to relatively precisely represent both numerical and non-numerical symbols increase with age. However, it was also found that 4-year-old children were capable of representing non-numerical symbols but not numerical symbols; 5-year-olds performed better at non-numerical symbols than numerical symbols, and this performance difference disappeared at the age of 6 years. After the age of 6 years, children performed better at representing numerical symbols than non-numerical symbols (Barth et al., 2005, 2008; Halberda et al., 2008; Kolkman et al., 2013; Lourenco, 2015; Matejko and Ansari, 2016; Lourenco and Bonny, 2017; Vanbinst et al., 2018). In line with the previous studies, our study revealed that, as age increases, both numerical and non-numerical symbol representation abilities are enhanced. Different from those previous studies, our study revealed that at the age of 4 years, Chinese children exhibit proficiency in representing numerical symbols, with the representation of numerical symbols better than that of non-numerical symbols. Both the initiation of numerical symbol representation and the turning point in the developmental trajectory, where the ability to represent numerical symbols surpasses that of non-numerical symbols, occur earlier in Chinese children than in their Western counterparts.

This difference may be attributed to the influence of cultural and education factors on the early development of numerical cognition (Siegler and Mu, 2008; Sarama and Clements, 2009; Muldoon et al., 2011; Laski and Yu, 2014; Xenidou-Dervou et al., 2015; Dowker and Li, 2019). For example, Siegler and Mu discovered that compared to their American counterparts, 5-year-old Chinese children exhibited lower PAE and linear representation of numerical quantities. They argued that Chinese children’s linear representation of numbers surpassed that of American children by 1–2 years. To further discern the roles of culture, family, and school education, Laski and Yu compared the NLE abilities of 6-year-old Chinese children with those of Chinese American children. Despite sharing the same culture and home numeracy environment, the Chinese American children differed in early school instruction from the Chinese children. The research findings indicate that a transparent counting system reflecting the decimal system contributes to understanding mathematical concepts, with both Chinese and Chinese American children surpassing the performance of other American children previously studied. Chinese children outperform other American children by 2 years and Chinese American children by 1 year. They suggested that school educational methods may exert a greater impact on mathematical development than the linguistic structure of the counting system. While Muldoon et al. (2011) found that Chinese children did not outperform British children in the NLE task, this was because they selected Chinese and British samples based on comparable abilities. When mathematical abilities, as measured by The British Ability Scales’ “Early Number Concepts” battery, were compared, Chinese children (mean age: 55 months; range: 47–62 months) were 10 months younger than British children (mean age: 64 months; range: 57–70 months). The NLE performance of both groups of children with comparable abilities was similar; however, this indicates that at a comparable chronological age, Chinese children outperform British children in NLE tasks. Additionally, a study conducted on 7-year-old children in Hong Kong, China, and Oxford, England, found no difference in PAE between the two groups, but the response time of the Hong Kong Chinese children was faster (Dowker and Li, 2019). This also demonstrated the influence of culture and education on children’s NLE.

We also found that the accuracy of the 4- and 5-year-old children’s NLE gradually increases in both numerical symbol and non-numerical symbol tasks, and the pattern of the 4- and 5-year-old children’s NLE is similar in numerical and non-numerical symbol tasks. Our results provide the same trajectories of numerical and non-numerical symbol representations. These results differ from those of Li et al. (2018), whose study found that at the age of 4 years, children possessed the ability to compare non-symbolic numbers, while at the age of 5 years, they exhibited the ability to compare numerical symbols, with non-numerical symbol representation superior to numerical symbol representation at this time. By the age of 6 years, the advantage of numerical symbol representation emerged. Similarly focusing on Chinese children, our study found that children already possessed relatively precise numerical symbol representation abilities at the age of 4 years, and at this time, they demonstrated superior non-numerical symbol representation. One possible reason for this difference may lie in the varying task difficulties between the two studies. Our study utilized numerical symbol and non-numerical symbol NLE tasks, focusing solely on the range of 0–10. In contrast, a study by Li et al. employed numerical magnitude comparison tasks with numbers ranging from 5 to 50, significantly increasing the difficulty level of the experimental tasks compared to our study. Furthermore, our study presents dots of consistent sizes, while the dots in Li et al.’s tasks vary in size, further increasing the relative difficulty of the numerical magnitude comparison tasks. Beyond that, the children in Li et al.’s study were from the Western region of China, while the children in our study were from the Eastern region of China, where the economy and basic education are more developed. Children from the Eastern region of China learn to perform even simple calculations at the age of 3 years.

Furthermore, we also found that the pattern of the number line for numerical symbol representation is similar to that of non-numerical symbol representation. Both 4- and 5-year-olds underestimated target numbers in the DP and NP tasks, whereas they overestimated target numbers in the PD and PN tasks. More importantly, children of both age groups had a similar pattern of estimation for both numerical and non-numerical symbols. The potential reasons for the distinct patterns observed in the DP vs. PD tasks and the NP vs. PN tasks may lie in differences in task complexity. The DP and NP tasks necessitate the translation between dots or numbers and spatial positions, posing a higher cognitive demand for young children. Previous research has found that children exhibit a trend of initially overestimating (with small numbers) followed by underestimating (with large numbers) in the NP task, while in the PN task, a mirrored pattern emerges, where children initially underestimate (with small numbers) followed by overestimating (with large numbers), as computed by the PAE. This study directly computed the PRE of estimation and found that in the NP task, children tend to underestimate, whereas in the PN task, they tend to overestimate. Although this study did not directly employ similar calculation methods for comparison, the mirrored relationship between the NP and PN tasks found in previous research indirectly supports the notion that the estimation patterns in these two tasks are exactly opposite (Siegler and Opfer, 2003; Iuculano and Butterworth, 2011; Ashcraft and Moore, 2012). Additionally, Iuculano and Butterworth also found that children took significantly longer to complete the PN task compared to the NP task, suggesting that the PN task imposes higher demands on children. This increased demand is likely to lead to underestimation.

By employing the NP, PN, DP, and PD tasks, we observed that Chinese children as young as 4 years already demonstrated relatively precise non-numerical and numerical symbol representations for magnitudes greater than 4, and the NLE patterns for 4- and 5-year-olds were similar in both numerical and non-numerical symbols. The onset and turning point in the developmental trajectory of numerical symbol representation, where the ability to represent numerical symbols surpasses that of non-numerical symbols, occurred earlier in Chinese children compared to their Western counterparts. While this study provides valuable insights into the development of numerical and non-numerical symbol representation among children, several limitations should be acknowledged. First, the lack of measurement of domain-general abilities (such as language, spatial ability, or executive function) in the study may pose a potential limitation. Additionally, this study cannot conclusively infer the reasons underlying the distinct estimation patterns observed in the DP vs. PD tasks and the NP vs. PN tasks. Future research could address these limitations by incorporating more measures of domain-general abilities and exploring additional factors that may influence numerical symbol representation in children. Furthermore, an exploration into the reasons behind the observed divergent patterns should be pursued.

## Data availability statement

The raw data supporting the conclusions of this article will be made available by the authors, without undue reservation.

## Ethics statement

The studies involving humans were approved by the Huzhou University Academic Ethics Committee. The studies were conducted in accordance with the local legislation and institutional requirements. Written informed consent for participation in this study was provided by the participants’ legal guardians/next of kin.

## Author contributions

ML: Conceptualization, Formal analysis, Funding acquisition, Methodology, Supervision, Writing – original draft, Writing – review & editing. JY: Data curation, Investigation, Writing – review & editing. XL: Data curation, Investigation, Writing – review & editing.
